# Factors associated with delayed diagnosis of tuberculosis in hospitalized patients in a high TB and HIV burden setting: a cross-sectional study

**DOI:** 10.1186/1471-2334-12-57

**Published:** 2012-03-15

**Authors:** Denise Rossato Silva, Alice Mânica Müller, Paulo de Tarso Roth Dalcin

**Affiliations:** 1 Faculdade de Medicina, Universidade Federal do Rio Grande do Sul (UFRGS), Hospital de Clínicas de Porto Alegre. Rua Ramiro Barcelos, 2350 - Largo Eduardo Zaccaro Faraco - Bairro Santa Cecília -Porto Alegre, RS, 90.035-903, Brazil

**Keywords:** Tuberculosism, Diagnostic delay, Treatment delay, Risk factors

## Abstract

**Background:**

The most essential components of TB control are early diagnosis and adequate treatment. Delay in the diagnosis and treatment of tuberculosis may result in more extensive disease and more complications, increase severity of the disease and is associated with higher risk of mortality. The purpose of this study was to identify factors associated with delayed diagnosis of TB in hospitalized patients.

**Methods:**

We conducted a cross-sectional study in a general, tertiary care, university-affiliated hospital. Adult patients with TB that were hospitalized were identified retrospectively, and risk factors for delayed diagnosis were collected.

**Results:**

The median delay until diagnosis was 6 days (IQR: 2-12 days). One hundred and sixty six (54.4%) patients were diagnosed ≤ 6 days, and 139 (45.6%) > 6 days after admission. The main factors associated with diagnostic delay (> 6 days) were extra-pulmonary TB and negative sputum smear.

**Conclusions:**

Although hospitalization permits a rapid management of the patient and favors a faster diagnosis, we found an unacceptable time delay before the diagnosis of pulmonary TB was made. Future studies should focus on attempt to explain the reasons of diagnostic retard in the patients with the characteristics related to delay in this study.

## Background

Tuberculosis (TB) is a major public health issue worldwide, particularly in low- and middle-income countries. It is estimated that one third of the world population is infected with *Mycobacterium tuberculosis *[1]. Brazil is ranked 19^th ^among the 22 high-burden countries that collectively account for 80% of TB cases globally, with an incidence of 37.9 cases/100,000 inhabitants/year in 2009 [2]. The city of Porto Alegre, in southern Brazil, has the highest incidence of TB (109.4 cases/100,000 inhabitants/year in 2010) and TB-HIV coinfection (35%) in the country [3].

The most essential components of TB control are early diagnosis and adequate treatment. It is estimated that a single infectious person who remains untreated can infect between ten and fifteen people every year, spreading the infection in the community [1,4]. In addition, delay in the diagnosis and treatment of tuberculosis may result in more extensive disease and more complications, increases severity of the disease and is associated with higher risk of mortality [5-7]. According to 2 studies, the main factors associated with diagnostic delay included HIV infection, negative sputum smear, extra-pulmonary TB, old age, and female sex [8,9].

Considering that 30-40% of all new cases of TB in Porto Alegre are notified in hospitals [10], it is important to find reasons for delay in in-hospital diagnosis of TB in order to develop new methods or protocols to reduce this delay. The purpose of this study was to identify factors associated with delayed diagnosis of TB in hospitalized patients.

## Methods

We conducted a cross-sectional study in a general, tertiary care, university-affiliated hospital with 750 beds, located in the city of Porto Alegre, Rio Grande do Sul State, in southern Brazil. Porto Alegre is the city with the highest incidence of TB in Brazil (115 cases/100,000 population), with a cure rate of 67%. Also, the TB-HIV co-infection rate is the highest in the country (35%) [3].

Adult patients (≥ 18 years old) with all forms of TB that were hospitalized during the period of three years (January 2008 - January 2011) were identified retrospectively. We included only the patients who began treatment for TB after hospitalization. Patients who were already receiving treatment at admission were excluded from this study. Pulmonary TB was diagnosed according to any of the following criteria established in the Brazilian Guidelines for Tuberculosis [11]: 1) detection by a direct test (Ziehl-Neelsen [ZN] method) - two positive samples; or 2) detection by a direct test (ZN method) - one positive sample and a positive culture result for *Mycobacterium tuberculosis *(in Löwenstein-Jensen [LJ] medium); or 3) detection by a direct test (ZN method) - one positive sample and radiological findings compatible with TB; or 4) only a positive culture result for *Mycobacterium tuberculosis *(in LJ medium); or 5) presence of clinical, epidemiologic and radiographic findings compatible with TB. The diagnosis of extra-pulmonary TB was based on clinical and/or complementary tests according to the location of TB [11]. In addition to the most common tests as acid-fast bacilli smear, culture, drug susceptibility test and tuberculin skin test, our study hospital has all the standard major equipment to conduct biomedical research studies (ultracentrifuges, HPLC, PCR, electrophoresis, cytometer, etc.). Induced sputum and Bronchoscopy Unit are also available. The specimens were sent to the laboratory as soon as they were collected. Typical turn-around time in our laboratory is 2 hours for direct tests and 4 weeks for culture. Patients initiate treatment in the same day results were confirmed.

The following data were collected from patient records using a standardized data extraction tool: demographic data (sex, age, race, years of schooling), behavioral data (smoking status, alcoholism, injection drug use), and medical history (clinical form of TB, symptoms at admission, methods of diagnostic, presence of comorbidities, prior TB treatment, drug regimen, interval from hospital admission until diagnosis, length of hospital stay, intensive care unit [ICU] admission, length of mechanical ventilation, hospitalization outcome [death or discharge], and outcome after discharge [cure, dropout, death]). The discharge policy is "as soon as possible", once patients are expected to be treated in outpatient's clinics. They remain hospitalized only if their clinical condition does not allow discharge. Data after discharge were obtained from SINAN (National System of Information on Notifiable Diseases). SINAN is a database from the Brazilian government which stores information concerning all notifiable infectious and contagious diseases. Also, we contacted by telephone the outpatient primary health care units where patients received the treatment after discharge, in cases without an outcome in this database. The duration of follow-up period was one year.

In order to evaluate the associated factors with delayed diagnosis, the study population was divided into two groups, according to the median time until diagnosis after admission to hospital: group 1 (≤ 6 days) and group 2 (> 6 days). The ethics committee at Hospital de Clínicas de Porto Alegre has approved access to patient records. Patient confidentiality has been maintained.

Data analysis was performed using SPSS 18.0 (Statistical Package for the Social Sciences, Chicago, Illinois). Data were presented as number of cases, mean ± standard deviation (SD), or median with interquartile range (IQR). Categorical comparisons were performed by chi-square test using Yates's correction if indicated or by Fisher's exact test. Continuous variables were compared using the *t*-test or Wilcoxon test. Multivariate logistic regression analysis was used to evaluate risk factors for delay, using selection of factors associated (p ≤ 0.10) with delay in univariate analysis or those known to have clinical significance. Hierarchical logistic regression models with predictors added one at a time were also examined to evaluate the possible collinearity among the predictors. The predictors selected in the final model were based on both numerical and clinical significance. The goodness of fit of the multiple logistic regression models was assessed using the Hosmer-Lemeshow test. Odds ratios (ORs) and nominal 95% confidence intervals (CIs) were presented. A two-sided p value < 0.05 was considered significant for all analyses.

## Results

Three hundred and five patients met the inclusion criteria and were included in the analysis. The characteristics of the study population are shown in Table 1. The median delay until diagnosis was 6 days (IQR: 2-12 days). According to time until diagnosis after admission to hospital, 166 (54.4%) patients were included in group 1 (≤ 6 days), and 139 (45.6%) in group 2 (> 6 days).

**Table 1:** Characteristics of study patients

Characteristics	Total n = 305	Group 1 (≤ 6 d) n = 166	Group 2 (> 6 d) n = 139	p value*
**Time until diagnosis (days)**	6 (2-12)	2 (1 - 4)	12 (9 - 19)	-

**Demographic characteristics**	41.9 ± 17.4	40.3 ± 16.1	43.8 ± 18.7	0.079
Age, yr				
Age > 60 years	40 (13.1)	17 (10.2)	25 (18.0)	0.051
Male sex	108 (35.4)	61 (37.7)	47 (33.8)	0.594
White race	230 (75.4)	123 (74.1)	107 (77.0)	0.560
**Current smokers**	91 (35.4)	56 (41.5)	35 (28.7)	0.032
**Alcoholism**	89 (29.2)	46 (27.9)	43 (30.9)	0.560
**Injection drug use**	81 (26.6)	50 (30.3)	31 (22.3)	0.116
**Forms of TB**				
Isolated Pulmonary TB	110 (36.1)	77 (46.4)	33 (23.7)	< 0.0001
Isolated Extra-pulmonary TB	143 (46.9)	53 (31.9)	90 (64.7)	< 0.0001
Pulmonary + extra-pulmonary TB	52 (17.0)	36 (21.7)	16 (11.5)	0.019
**Symptoms**				
Cough	132 (43.3)	89 (53.6)	43 (30.9)	< 0.0001
Night sweats	74 (24.3)	54 (32.5)	20 (14.4)	< 0.0001
Fever	190 (62.3)	111(66.9)	79 (56.8)	0.072
**Laboratory results**				
Smear-negative sputum	93 (56.7)	45 (42.1)	48 (84.2)	< 0.0001
**Radiographic patterns**				
Cavitary disease	33 (10.8)	28 (16.9)	5 (3.6)	< 0.0001
Pleural effusion	58 (19.0)	24 (14.5)	34 (24.5)	0.027
Miliary pattern	37 (12.1)	29 (17.5)	8 (5.8)	0.002
Consolidation	52 (17.0)	31 (18.7)	21 (15.1)	0.409
Normal	42 (13.8)	14 (8.4)	28 (20.1)	0.003
**Previous default from TB treatment**	24 (7.9)	20 (12.0)	4 (2.9)	0.003
**Comorbidities**				
Presence of any comorbidity	224 (73.4)	130 (78.3)	94 (67.6)	0.035
HIV positive	191 (71.0)	117 (75.5)	74 (64.9)	0.059
Diabetes mellitus	19 (6.2)	3 (1.8)	16 (11.5)	< 0.0001
Chronic renal failure	8 (2.6)	1 (0.6)	7 (5.0)	0.016
**Mechanical ventilation**	48 (16.3)	28 (17.4)	20 (15.0)	0.587
**In-hospital mortality**	50 (16.4)	29 (17.5)	21 (15.1)	0.579
**Mortality after discharge**	47 (18.4)	22 (16.1)	25 (21.2)	0.292

Continuous variables (age) are presented as mean ± SD; other data are presented as n/N (%): number of cases with characteristic/total number of cases (percentage in the group), or median (interquartile range)

* p for the comparison between groups 1 and 2

**Table 2:** Multivariate analysis of risk factors for delayed diagnosis

Characteristics	OR (95%CI)	p
Smear-negative sputum	4.71 (1.55 - 14.33)	0.006
Isolated extra-pulmonary TB	3.27 (1.26 - 8.49)	0.015

Current smoking was more common in group 1 (56 [41.5%]) than in group 2 (35 [28.7%]) (p = 0.032). Pulmonary TB was more frequent in group 1 (77 [46.4%]) than in group 2 (33 [23.7%]) (p < 0.0001), as well as extra-pulmonary TB was more common in group 2 (90 [64.7%]) than in group 1 (53 [31.9%]) (p < 0.0001). In addition, smear-negative cases were more prevalent in the group of patients diagnosed a week or more after admission to hospital (48 [84.2%] vs 45 [42.1%]; p < 0.0001). Patients with sputum smear negative or not performed (didn't produce sputum) were diagnosed using other tests like positive acid-fast bacilli in induced sputum or bronchoalveolar lavage, PCR, presence of granuloma and positive acid-fast bacilli in biopsy specimens, and culture. Only 32 patients were diagnosed exclusively by culture. There were no significant differences between the 2 groups in sex, race, need of mechanical ventilation, and mortality.

Cough and night sweats were more common in patients with a diagnosis in less than 6 days (89 [53.6%] and 54 [32.5%], p < 0.0001 for both). Cavitary disease and miliary pattern on chest X-ray were more frequent in group 1 as compared with group 2 (28 [16.9%] in group 1 vs 5 [3.6%] in group 2; p < 0.0001 and 29 [17.5%] in group 1 vs 8 [5.8%] in group 2; p = 0.002, respectively). On the other hand, the most prevalent radiographic patterns in group 2 (> 6 days) were pleural effusion (34 [24.5%]; p = 0.027) and a normal chest X-ray (28 [20.1%]; p = 0.003). All patients with normal chest X-ray had a diagnosis of isolated extra-pulmonary TB (data not shown).

A previous history of TB treatment default was more common in group 1 (20[12.0%]) than in group 2 (4 [2.9%]) (p = 0.003). Additionally, diabetes mellitus and chronic renal failure were more frequently present in those patients who were diagnosed after 6 days from hospital admission (Table 1). However, the number of patients with chronic renal failure was too small.

Logistic regression analysis estimating the ORs of risk of delayed diagnosis was conducted. Age, current smoking, isolated extra-pulmonary TB, cough, night sweats, fever, smear-negative sputum, cavitary disease, milliary pattern, pleural effusion, previous default from TB treatment, and patients with HIV, diabetes mellitus and chronic renal failure were included in the multivariate analysis. The Hosmer-Lemeshow test showed that the multivariate models demonstrated a good fit (p = 0.297), and there was no evidence for collinearity among the independent variables. The following variables remained significant in the final multivariate model: isolated extra-pulmonary TB (OR 3.27, 95% CI 1.26 - 8.49; p = 0.015) and smear-negative sputum (OR 4.71, 95% CI 1.55 - 14.33; p = 0.006).

## Discussion

In this cross-sectional study, we found that a median time from admission until diagnosis was 6 days, with 33.2% of patients being diagnosed before 6 days, and 45.6% in more than 6 days. The main factors associated with diagnostic delay were extra-pulmonary TB and negative sputum smear.

There is no agreed definition of what constitutes an "acceptable" delay. Ward and others [12] suggests that this interpretation should depend on the local health services and the local epidemiological situation, with a shorter delay to be expected when incidence is high. In a systematic review of literature [13], the reported ranges of average (median or mean) health system delay were 2-87 days for both low and high income countries. However, considering only in-hospital studies, the median interval from admission to initiation of TB treatment is in agreement with our results, varying from 4 to 12.5 days [14-18].

Despite a high incidence of TB, as suggested by Ward and others [12], 45.6% of patients in our study had diagnosis delayed by more than a week. In a population-based study in Canada, treatment was initiated after a week or more in 30% of all patients with pulmonary TB [14], which could be attributed to the relative rarity of active TB in this country, resulting in a lack of awareness of TB. However, in our study delayed diagnosis was related to extra-pulmonary TB and smear-negative pulmonary TB, which frequently carry diagnostic difficulties. In accordance with other studies [9,16,18-21], extra-pulmonary TB, including pleural TB, and smear-negative pulmonary TB cases were found to be associated with diagnostic delay. A cross-sectional survey of newly identified adult patients with TB demonstrated that patients with extra-pulmonary TB or smear-negative disease are significantly more likely to be hospitalized and are also more likely to have experienced treatment delay [21]. In a referral hospital in Rwanda, smear-negative pulmonary TB and extra-pulmonary TB were risk factors for a longer health system delay [18].

Current smoking was associated with an earlier diagnosis in our study. According to previously described in literature [22-24], smoking TB cases were significantly more likely to present with a cough and difficult or labored breathing, and less likely to present with only extrathoracic TB than never-smoking TB cases. They were significantly more likely to have upper-zone involvement, cavitation, positive bacilloscopy and sputum culture, and pulmonary TB than never-smoking TB cases.

Our analysis revealed that the presence of cough was associated with a faster diagnosis. In a retrospective study conducted in an emergency department, the presence of cough was related to more rapid isolation and treatment [25]. However, in other studies [9,26,27] cough, especially chronic, is observed as a cause of late presentation of patients to the health system. Nevertheless, once patients are in the health care system, this kind of complaint may give rise to earlier suspicion of TB, and consequently, earlier diagnosis and treatment.

We also found that the complaint of night sweats was more frequent in the group of patients diagnosed less than 6 days after admission. In a cross sectional hospital based survey in Tanzania [8], not knowing that night sweat is one of the TB symptoms was associated with patient delay in females. Regarding to our finding, it is possible that the presence of night sweats raise awareness of TB among health care workers.

Cavitary disease and milliary pattern on chest x-ray were associated with an earlier diagnosis in our investigation. A similar study has also shown that radiographic changes typical of TB, like apical infiltrates and cavitation, were previously associated with more rapid treatment [25].

Patients with HIV infection included in our study experienced some delay in diagnosis of TB, although this finding was not statistically significant. HIV is known to delay TB diagnosis due to non-specific results and atypical clinical presentation [28,29]. However, our findings are in agreement to other studies [5,30,31] that found no association between HIV positivity and delayed diagnosis. In a previous investigation, HIV-positive TB patients suffered more serious symptoms when TB was diagnosed, which prompted them to visit the hospital and increased the suspicion of TB by the clinician [31].

Our data did not show an increased mortality in patients with retard in diagnosis. Controversy still exists on the role of delay in TB diagnosis and treatment in the mortality rate. Although some authors demonstrated an association between diagnosis delay and mortality [32-35], in other studies delayed diagnosis was not a risk factor for mortality [16,33,36].

One of the limitations of this study is that we evaluated only health system delay, and not patient delay. Furthermore, patients were recruited from a single hospital, and the results may thus not apply to other settings. Besides, retrospective design has inherent limitations. The information obtained retrospectively from chart review is probably not as complete and accurate as when data collection is done prospectively. In spite of these concerns, the study of factors associated with delayed diagnosis is important as it has an impact in transmission dynamics of TB. Therefore, to identify the sources of delays is a critical issue for an effective TB control program.

In conclusion, we demonstrated that the median delay in TB diagnosis in this setting is 6 days, and the factors associated with this delay in multivariate analysis were extra-pulmonary TB and negative sputum smear. Reducing these delays may require increase of diagnostic awareness in health care professionals, and a review of health service practices. Future studies should focus on attempt to explain the reasons of diagnostic retard in the patients with the characteristics related to delay in this study. In addition, studies on health care seeking may be warranted in this setting.

## Competing interests

The authors declare that they have no competing interests.

## Authors' contributions

DRS participated in the conception of the study, in its design, collected the data, performed the statistical analysis and wrote the manuscript. AMM collected the data and helped to draft the manuscript. PTRD participated in the conception of the study, in its design, performed the statistical analysis and helped to draft the manuscript. All authors read and approved the final manuscript.

## Funding source

FIPE - Hospital de Clínicas de Porto Alegre.

## Pre-publication history

The pre-publication history for this paper can be accessed here:

http://www.biomedcentral.com/1471-2334/12/57/prepub
